# Feasibility of a Novel Functional Sensibility Test as an Assisted Examination for Determining Precision Pinch Performance in Patients with Carpal Tunnel Syndrome

**DOI:** 10.1371/journal.pone.0072064

**Published:** 2013-08-20

**Authors:** Hsiu-Yun Hsu, Li-Chieh Kuo, Yao-Lung Kuo, Haw-Yen Chiu, I-Ming Jou, Po-Ting Wu, Fong-Chin Su

**Affiliations:** 1 Department of Biomedical Engineering, National Cheng Kung University, Tainan, Taiwan; 2 Department of Physical Medicine and Rehabilitation, National Cheng Kung University Hospital, College of Medicine, National Cheng Kung University, Tainan, Taiwan; 3 Department of Occupational Therapy, National Cheng Kung University, Tainan, Taiwan; 4 Section of Plastic Surgery, Department of Surgery, National Cheng Kung University, Tainan, Taiwan; 5 Department of Orthopedics, National Cheng Kung University, Tainan, Taiwan; 6 Medical Device Innovation Center, National Cheng Kung University, Tainan, Taiwan; National Yang-Ming University, Taiwan

## Abstract

To understand the feasibility of a novel functional sensibility test for determining precision pinch performance in patients with carpal tunnel syndrome, this study investigates the validity, sensitivity and specificity of functional sensibility derived from a pinch-holding-up activity (PHUA) test. Participants include 70 clinically defined carpal tunnel syndrome (CTS) patients with 119 involved hands and 70 age- and gender-matched controls. To examine the discriminating ability of the functional sensibility test, the differences in the ability of pinch force adjustments to the inertial load of handling object between CTS and control subjects are analyzed. The results of functional sensibility are correlated with the severity of CTS to establish concurrent validity. The receiver operating characteristic (ROC) curve is constructed to demonstrate the accuracy of the proposed test. The functional sensibility score significantly discriminates the patients and control groups (respectively, 12.94±1.72 vs. 11.51±1.15N in peak pinch force (FP_Peak_), p<0.001; 2.92±0.41 vs. 2.52±0.24 in force ratio, p<0.001) and is moderately correlated (*r* = 0.42–0.54, *p*<0.001) with the results of traditional sensibility tests (touch-pressure threshold and two-point discrimination test). In addition, there is a statistical difference in the results of functional sensibility (p<0.001) among the subgroups of CTS severity based on electrophysiological study. The sensitivity and specificity are 0.79 and 0.76, respectively, for the functional sensibility test. The areas under the ROC curve are 0.85 and 0.80 for the force ratio and FP_Peak_, respectively. In conclusion, the functional sensibility test could be feasibly used as a clinical tool for determining both the sensibility and precision pinch performance of hands for the patients with CTS.

## Introduction

Carpal tunnel syndrome (CTS), the most common peripheral neuropathy in upper extremities, is usually caused by mechanical or dynamic compression for the median nerve [1]. In the early stages of CTS, patients typically have sensory disturbance such as nocturnal pain, paresthesia, and numbness involving the median-innervated territory, with motor weakness being relatively rare [2]. However, a study has found that sensory disturbance leads to a discrepancy between median nerve motor function and motor skills in CTS patients [3]. Thus, momentary sensory information is required for optimal motor control.

Electrophysiological testing is recommended as the gold standard and important for assisting clinicians in determining median nerve compression in the area of the carpal tunnel, especially for patients with atypical symptoms. In addition, nerve conduction study (NCS) has been proposed as the simplest and least uncomfortable test used for obtaining information about median nerve function [4]. Grades for CTS hands based on NCS findings are useful for clinicians to understand the severity of median nerve compression [5]. With regard to the assessment of sensory function in patients with carpal tunnel syndrome, the sensory nerve conduction velocity, Semmes-Weinstein monofilament (SWM), and two-point discrimination (2PD) tests [1], [6] are the most commonly used. Notably, threshold testing has been reported to be reliable for quantifying sensory function for CTS [7]. However, there is no direct relationship between the discriminative sensibility and the clinical test results for CTS [8]. Some researchers have indicated that specific impairments in sensibility and functional capability of the hand are weakly linked in nerve injury patients [9], [10]. Therefore, Moberg (1958) redefined hand function as a sensory grip, and then developed a picking-up test to determine functional sensibility in the hand following reconstructive hand surgery [10]. Numerous sensory tests have been developed for determining manual functional deficits for the impaired-sensation population [11], [12]. Nevertheless, most of these tests do not determine the quality of movement control in the hand. To determine the characteristics of sensori-motor control in the hand, the precision grip has been used as an experimental model to investigate how cutaneous sensory information contributes to functional performance [13], [14], [15], [16], [17], [18]. The results suggest that a partial impairment of tactile sensibility affects motor efficiency in a pinch-lifting task.

Hand dexterity is compromised in the early stages of carpal tunnel syndrome [19], [20]. Sensory-deficient CTS patients are reported to have problems efficiently generating grip forces when using hand tools [21]. In contrast, Thonnard et al. indicated that impaired sensibility did not significantly impact the efficiency of generating grip forces for eleven CTS patients [22]. However, the numbers of recruited subjects in these studies are insufficient for drawing definitive conclusions. Moreover, Cole et al. used an external compression over the carpal canal to reduce the median nerve function in healthy adults [23] and found an elevated pinch force when executing tasks is associated with insufficient sensory information. Nevertheless, the patients with prolonged peripheral nerve compression may have long-lasting modification of the cortical neural representation [24], [25]. Therefore, the results obtained from subjects after induced impaired sensation might not actually represent the same or similar performance as the CTS patients did.

The precision pinch performance has recently been analyzed to help the clinicians to understand how the sensory function affects the control of muscular responses in the hand. The ability of pinch force adjustment to the inertial load changes in a pinch-holding-up activity (PHUA) test was defined as an index of functional sensibility in our previous effort, while the test was shown to be highly reliable [26]. Nevertheless, the validity and accuracy of the test for the CTS patients has not been proved. Hence, the first aim of the present study is to examine the usefulness of a functional sensibility test in differentiating well-defined CTS patients from healthy subjects and distinguish among subgroups with various CTS severities. The second aim is to demonstrate the concurrent validity of the test. The relationship between the functional sensibility and traditional sensibility (SWM and 2PD) was examined based on a large sample size of CTS and healthy subjects. In addition, to understand the accuracy of the proposed evaluation method for diagnosing CTS, the third aim is to construct the sensitivity and specificity of this novel functional sensibility test.

## Materials and Methods

### Subjects

Study participants included clinically defined CTS patients and healthy control subjects. For the CTS patients, the diagnosis was based on clinical tests, sensibility tests, and nerve conduction studies, and the inclusion criteria were: (1) significant sensory symptoms of CTS; (2) lesions involving the distal median nerve, as found using an electrophysiological test; (3) patients with good-to-normal grade of muscle power of abductor pollicis brevis muscle; (4) no diabetic mellitus, other neurological deficits, or previous hand injuries; and (5) right-handedness. Seventy patients (54 females and 16 males) with CTS were referred by rehabilitation medicine and orthopaedic outpatient clinics during the period of March 2010 to September 2010. The age range was from 31 to 77 years, and the mean age was 53.84±10.82 years. In addition, the same numbers of age-, gender-, and handedness-matched healthy controls were recruited through posting advertisement from the local community. Control subjects were confirmed by the absence of sensori-motor disturbance in the hand or any neurological disorder affecting the hand. The demographic data of the participants are shown in Table 1. The recruited subjects, 21 patients with uni-lateral CTS and 49 patients with bilateral CTS, exhibited classic CTS in 119 hands (64 right hands and 55 left hands).

**Table 1:** Demographic data of patient and control groups.

	Patient group (n* = 119)	Control group (n* = 119) *p*-value	
**Demographic data**	Male : Female = 16∶ 54	Male : Female = 16∶ 54	
Gender			
Age (years)	53.84±10.82	53.69±10.79	
Dominant : Non-dominant hand	64∶ 55	64∶ 55	
**Traditional sensibility assessments**			
S2PD (mm)	5.1±1.5	3.7±0.7	<0.001
M2PD (mm)	4.3±1.4	2.9±0.6	<0.001
SWM test	3.45±0.38	2.89±0.27	<0.001
Stress (gm/mm^2^)	15.36±7.92	4.19±0.82	<0.001

* n indicates the number of affected hands. Forty-nine patients had CTS in both hands. Seventy CTS patients had a total of 119 affected hands.

Statistical analysis method: Mixed effect model; the significance threshold was set at 0.05.

### Ethics Statement

All participants were informed about the purpose of the study and signed consent forms. The study was approved by the Institutional Review Board (No. ER-98-257) of National Cheng Kung University Hospital.

## Instruments

### 

#### Nerve conduction velocity

The sensory and motor NCS were performed on the patients using a Medelec Synergy N-EP EMG apparatus (Oxford Instruments Medical, Inc., Tubney Woods, Abingdon, Oxon, UK). Electrophysiological data were measured from 119 affected hands of the patients. The obtained data were used to reflect the condition of the median nerve for the patients and were compared with reference values from healthy subjects [27]. The grading of median nerve impairment for CTS was according to the classification of the electrophysiological study reported by Padua et al. [5]. The severity of CTS was divided into 6 grades: grade 1 indicates normal sensory or motor response, grade 2 indicates abnormal response in segmental or comparative tests only (minimal); grade 3 indicates abnormal digit-wrist sensory nerve conduction velocity and normal distal motor latency (mild), grade 4 indicates abnormal digit-wrist sensory nerve conduction velocity and abnormal distal motor latency (moderate), grade 5 indicates absence of sensory response and abnormal distal motor latency (severe), and grade 6 indicates the absence of motor and sensory responses (extreme).

#### Sensibility tests

The sensibility tests used to evaluate the sensory status of the hands of recruited subjects included static-2PD (S2PD), moving-2PD (M2PD), and Semmes-Weinstein (SW) monofilament tests. Two point discriminative tests were used to detect the least distance that the subjects could perceive. The S2PD and M2PD test the innervation density of slowly and quickly adapting nerve fibers, respectively, and their corresponding mechanoreceptors. The SWM test is used to determine the touch-pressure threshold of the hands. When applied, the filament exerts a constant force onto the skin area. SW monofilaments are labeled with a numerical marking which is a log to the base ten of the force in tenths of milligrams [28], [29].

#### Pinch apparatus

The pinch apparatus (weight = 480 g) was designed in our previous work [30]. It consists of two 6-axis load cells (force/torque transducers: Nano-25; ATI Industrial Automation, Apex, NC) that register the online pinch force exertion by the subjects and an accelerometer (Model 2412; Silicon Designs, Inc., Issaquah, WA) that detects the acceleration of the pinch apparatus in space. The obtained intra-correlation coefficient values ranged from 0.84 to 0.96 of the functional sensibility derived from the PHUA test, indicating a high reliability of repeated measurements.

#### Procedures of the PHUA test (Fig. 1A)

Before executing the PHUA test, the subjects first washed their hand with soap and water to remove all greasy substances. The subjects then sat upright with their unsupported forearm extending forward. They were asked to pinch and lift the pinch device using the pulps of the thumb and index finger to about 5 cm above the table, and then hold this position for 5 seconds (static holding phase). Afterwards, they lift the apparatus to a height of 30 cm (lifting phase) and then slowly lower it to its initial position. The data collection period for each trial was 15 seconds. Verbal instructions regarding the timing of task sequences were given to the subjects. In addition, they were instructed to lift the apparatus gently with a self-paced lifting speed prior to the experiment. The participants were allowed three practice trials to ensure that they completely understood the PHUA test before formal data recording. The test procedures were repeated three times for each hand, with a 10-min resting interval between each trial.

#### Data processing and analysis

**Figure 1:**
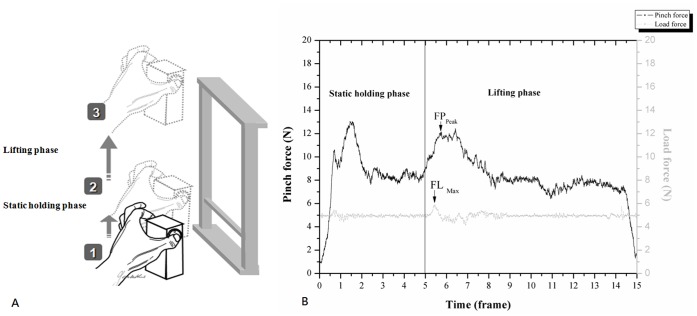
The experimental procedures and data analysis of this study. (A): Schematic representation of the pinch-holding-up activity. (B): Data analysis of a functional sensibility test.

The following functional sensibility parameters were used in the analysis of a PHUA task (Fig. 1B): (1) FP_Peak_: maximum pinch force during the lifting phase in the PHUA test; (2) FL_Max_: maximum load force at maximum upward acceleration onset; and (3) force ratio: the ratio between FP_Peak_ and FL_Max_. A custom-made Matlab program was used to compute these parameters. The discriminative sensory function was assessed by determining the minimum distance which the subjects could detect in the static and moving 2PD tests. The numerical markings and stress (force per area) determined from the SW monofilament test was defined as pressure threshold of hands. The adopted electrophysiological classification was used as the severity of impairment in the median nerve function for the CTS patients.

#### Experimental protocol

Basic personal information, the force parameters obtained from the PHUA test, and the results of traditional sensibility tests were recorded for all participants. Electrodiagnostic studies were carried out for all CTS subjects.

#### Statistical analysis

SAS 9.3 for Windows (SAS Institute Inc., Cary, NC, USA) was used for statistical analysis. Descriptive statistics were used to describe a mean and standard deviation of the dependent variables, namely the results of the SW monofilament test, S2PD, M2PD, and parameters (force ratio and FP_Peak_) of the functional sensibility test. Nevertheless, 49 of the recruited CTS patients had bilateral hands assessed which might affect the assumption of independent observations within the samples chosen in this study. Therefore, for the dealing with the random effect of dependency of observation, a mixed effect model was used to analyze the difference in the functional and traditional sensibility test results between CTS and healthy control group and among CTS subgroups of different severity based on the NCS findings. A statistically significant difference in testing results was indicated by *p*<0.05. In addition, Bonferroni multiple comparisons were used to analyze the differences in outcome variables between each pair of the CTS subgroups. According to Bonferroni’s correction, the threshold of significance was at *p* = 0.008. The correlations between parameters of force ratio and FP_Peak_ and sensibility test scores were calculated using Pearson’s correlation coefficient with a statistical significance threshold of 0.05. The receiver operating characteristic (ROC) curve was constructed using sensitivity and specificity values from results of functional sensibility derived from the PHUA test of the patient and healthy groups to determine the cutoff value. To determine the accuracy of the PHUA test, the area under the ROC curve (AUC) was calculated.

## Results

### Discriminative Validity

**Figure 2:**
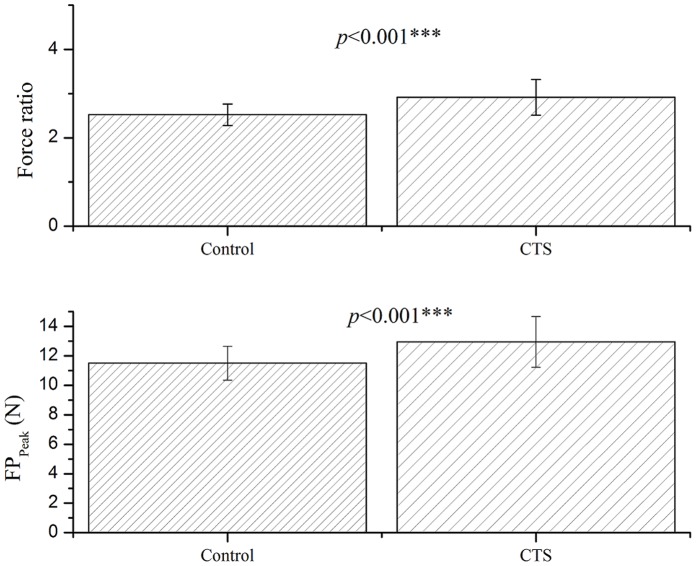
Mean (±SD) of force ratio (upper) and FP_Peak_ (lower) for CTS and healthy groups. Statistical differences were observed in both the force ratio and FP_peak_ between CTS and control groups. (Statistical analysis method: mixed effect model; the level of significance was set at *p*≤0.05).

The results show statistically significant differences of sensory status (Table 1) and functional sensibility (ability of responding pinch force to actual load of the handled object) between the CTS and control subjects (respectively, 12.94±1.72 vs. 11.51±1.15N in FP_Peak_, *p*<0.001; 2.92±0.41 vs. 2.52±0.24 in force ratio, *p*<0.001) (Fig. 2).

### Concurrent Validity

**Figure 3:**
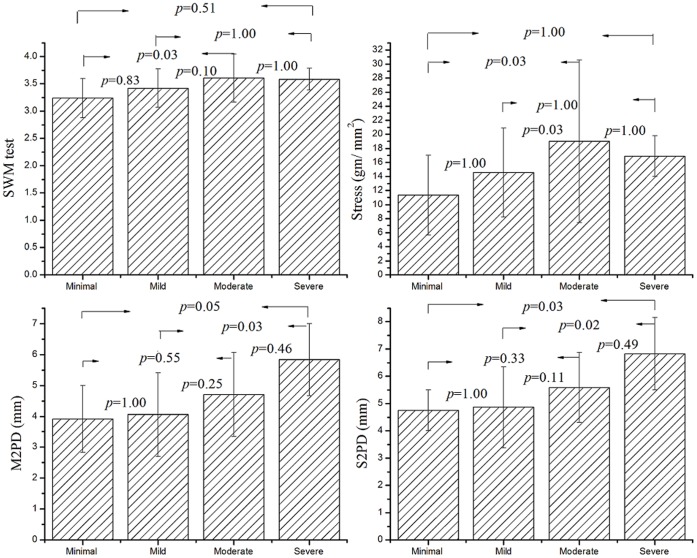
Mean (±SD) of SWM, M2PD, S2PD and stress determined from SWM result for various grades of CTS severity based on nerve conduction study. The difference in all of the traditional sensibility tests between each pair of the CTS subgroups did not reach statistical significance. (Statistical analysis method: the mixed effect model with Bonferroni multiple comparisons test; the significance threshold analyses for Bonferroni post hoc test was set at *p* = 0.008.).

**Figure 4:**
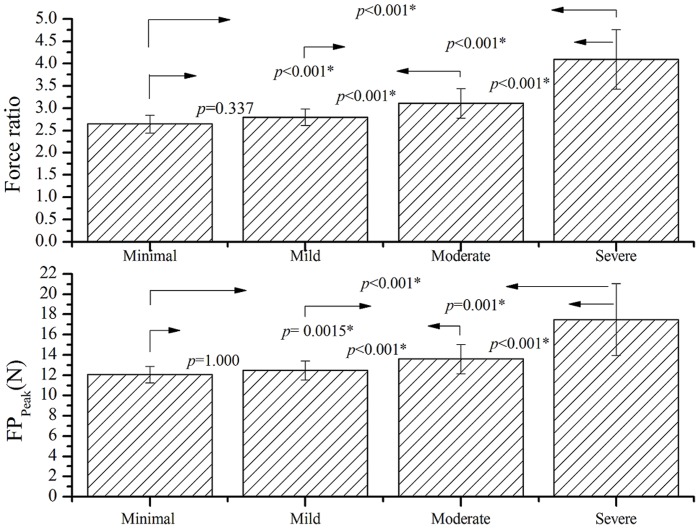
Mean (±SD) of force ratio and FP_Peak_ for various grades of CTS severity based on nerve conduction study. There were statistical differences in the results of functional sensibility between each pair of the CTS subgroups except between the subgroup with minimal and mild severity. (Statistical analysis method: the mixed effect model with Bonferroni multiple comparisons test; the significance threshold for Bonferroni post hoc test was set at *p* = 0.008.).

Table 2 shows that there is a moderate relationship between the two parameters of functional sensibility (FP_Peak_ and force ratio) and the results of traditional sensibility tests (*r* = 0.42–0.54, *p*<0.001). The differences of functional sensibility and traditional sensibility measured from the patients among the different grades of CTS severity based on electrophysiological study are revealed in Fig. 3 and Fig. 4. The results of SWM test are 3.23±0.36, 3.42±0.35, 3.61±0.44 and 3.58±0.20, M2PD are 3.9±1.1, 4.1±1.4, 4.7±1.4 and 5.8±1.2 mm and S2PD are 4.8±0.8, 4.9±1.5, 5.6±1.3 and 6.8±1.3 mm for the CTS subgroups with minimal, mild, moderate, and severe grade, respectively. The difference in each of traditional sensibility tests between the CTS subgroups with different severity (Fig. 3) did not reach the statistical significance (*p*>0.008). The force ratios are 2.64±0.20, 2.79±0.19, 3.11±0.33, and 4.10±0.67 and the FP_Peak_ values are 12.08±0.81, 12.47±0.94, 13.61±1.45, and 17.48±3.53 N for the subgroups with minimal, mild, moderate, and severe grade CTS, respectively. The results examined by a mixed model approach show that there were statistical differences (*p*<0.001) in the results of functional sensibility between each pair of the subgroups (Fig. 4) except that the difference between the subgroups with minimal and mild CTS did not reach the significance level in both the parameters of FP_Peak_ and force ratio (*p*>0.05). In addition, the functional sensibility test has the statistical power greater than 0.9 for detecting the difference in the force ratio and FP_Peak_ between the CTS and controls or between the subgroups of CTS severity according to the power calculation by the mixed effect model.

**Table 2:** Correlation of traditional hand sensibility tests and parameters of pinch force adjustment in PHUA test for CTS patients.

	Force ratio	FP_Peak_	SWM test	Stress	M2PD	
FP_Peak_	0.91*					
SWM test	0.54*	0.48*				
Stress	0.56*	0.50*	0.96*			
M2PD	0.50*	0.44*	0.45*	0.47*		
S2PD	0.48*	0.42*	0.41*	0.44*	0.90*	

Statistical analysis method: Pearson’s correlation coefficient; the significance threshold was set at 0.05.

* *p*<0.001.

### ROC Curve of Functional Sensibility

**Figure 5:**
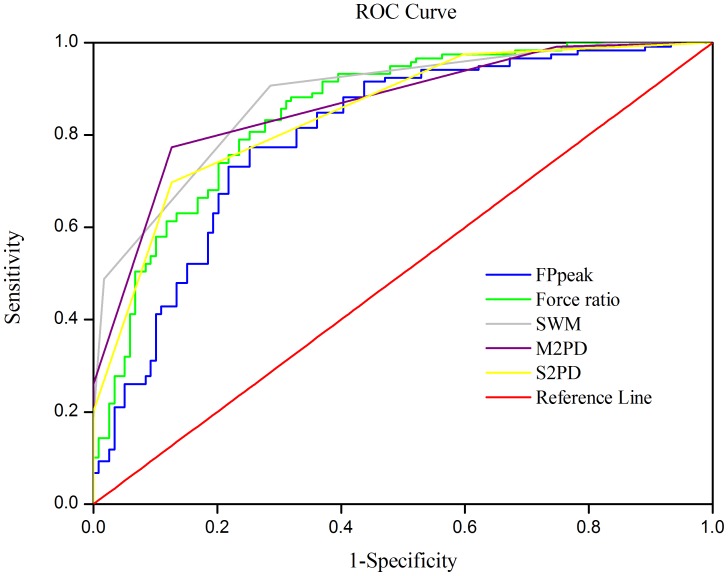
Constructed ROC curves for functional sensibility and traditional sensibility tests.

Sensitivity, specificity values, and optimal cutoff point calculated for functional sensibility and traditional sensibility tests are shown in Fig. 5. The sensitivity was 0.79 and specificity was 0.76 for the force ratio in the PHUA test. The results suggest a score of 2.66 for force ratio and 12.00 for FP_Peak_ as the optimal cutoff point when screening for CTS patients. According to the AUC of the functional sensibility parameters (FP_Peak_ = 0.800 and force ratio = 0.851), the PHUA test is accurate for diagnosing CTS. Compared to traditional sensibility tests, the area under the curve of the force ratio is slightly lower than the areas of SWM (0.882) and M2PD (0.865) but higher than the area under the S2PD (0.849).

## Discussion

Standardized measurements of sensory function for CTS patients can generally be classified into the threshold test, the discriminative sensory test, and electrodiagnostic study. However, the obtained results of these standardized tests are inadequate for identifying the hand function of sensory-deficient patients. Though Moberg have developed a pick-up test to test the sensory-grip ability for the reconstructive hands, the quality of movement was difficult to be detected from the test. Recently, the combined uses of the rapid pinch and release psychomotor test with the gap detection sensory test, which was designed to monitor functional deficits associated with CTS, has been proved to have sufficient sensitivity and specificity for differentiating CTS patients from healthy subjects in previous studies [31], [32]. Nevertheless, the small sample size and subject selection spectrum bias was the limitation of these studies. Our previous works have developed a reliable apparatus and method for analyzing functional sensibility in patients with nerve injury [26], [30], [33], with results showing that improved sensory function helped restore patients’ performance in precise pinch force control evaluations. Moreover, the efficiency of prehensile forces in a voluntary lifting task is automatically adjusted by pre-programmed muscle commands according to somatosensory information, and not the conscious control of the subjects [34]. Therefore, the ability to adjust the pinch force according to the actual load of the handled object could reflect the characteristics of the sensori-motor system of a hand when executing a basic pinch-lifting task.

To validate functional sensibility derived from a PHUA test, the sensory status and precision grip ability responding to load fluctuations induced by momentum-change were analyzed for CTS and healthy subjects in this study. Decreased sensibility and inefficiency in the interactive pinch force were found in the CTS group. The higher pinch force output for the CTS patients compared with that of healthy subjects indicates that sensory symptoms affect the motor control ability of the hand. Additionally, the force ratio and FP_Peak_ has greater than 90% power for detecting difference either between CTS and healthy subjects or the subgroups of CTS was in this study. The result revealed that that the PHUA test is a useful tool for detecting deficit in precision pinch performance for CTS patients. In this study, 86 hands were had either minimal or mild CTS and 33 hands had either moderate or severe CTS. Hence, nearly 3/4 of the patients had only sensory symptoms; however, most of them had problems in the efficiency of movement. The obtained results show that a decline in the effective performance of CTS subjects is associated with sensory function. According to Fitts’ (1954) model of skill acquisition, the uniformity of motor performance is based on the human motor capacity plus relative sensory feedback [35]; therefore, normal sensory feedback from the hands provides sufficient information for optimizing the forces in the PHUA test. That might explain why the sensory symptoms of CTS subjects lead to a discrepancy between the motor function of the median nerve and motor execution [3].

The present study demonstrated that there is a moderate correlation (*r* = 0.42–0.54, *p*<0.001) with statistical significance between the force ratio and traditional sensibility tests (SWM and M2PD tests). This indicates a significantly moderate agreement between the functional sensibility and traditional sensibility tests, and that the test can be used to determine sensation ability in the hand. The results of the functional sensibility test for CTS subjects with various grades of median nerve impairment were analyzed. There was a statistical decline in efficiency of adjusting force output with increasing severity of CTS based on the results of an electrophysiological study. Clinically, the amplitude of sensory nerve action potentials (SNAP), the peak distal latency of SNAP, and the conduction velocities in the wrist-to-palm segment obtained from a nerve conduction test help clinicians determine the actual nerve function, which is related to the severity of compression on the median sensory nerve. Therefore, the results of the functional sensibility test reflect the actual functional status of the median nerve. The force ratio did not show significant difference in the minimal and mild subgroups (*p* = 0.337), which might be due to only 12 affected hands being in the minimal group, and thus there was insufficient separation between the two subgroups.

The index used for evaluating diagnostic accuracy is the AUC [36]. The AUCs for the force ratio and FP_Peak_ are 0.851 and 0.800 respectively. The functional sensibility derived from the PHUA test is as good at separating CTS subjects from healthy subjects as are the standardized sensory tests. In addition, both parameters of functional sensibility had high sensitivity and specificity for diagnosing CTS. Where the best sensitivity-to-specificity was obtained, scores of 2.66 for force ratio and 12.00 for FP_Peak_ were determined as the optimal cutoff point for screening CTS. Previous publications [37], [38] reported the gold standard methodology, sensory nerve conduction study, has a sensitivity ranging from 67to 85 percent and a specificity ranging from 58 to 98 percent for CTS. Compared to sensitivity and specificity of the reference gold standard, the novel functional sensibility test revealed the accuracy in assessing CTS. The results also indicated that the SWM, M2PD, and S2PD tests had high accuracy for discriminating CTS. Nevertheless, the reference values for impaired sensibility obtained using the M2PD and S2PD tests were 4 and 6 mm, respectively [29]; the obtained cutoff values of M2PD and S2PD for CTS were 3.5 and 4.5 mm, respectively, in this study. That is, even if patients have severe sensory symptoms, the 2PD test results will be within the normal range in a large portion of CTS patients. The resolution of the discriminative sensory test is limited for studying sensory function for CTS. Clinicians should thus use the optimal cutoff values of functional sensibility in combination with the results of threshold sensory tests to accurately determine peripheral neuropathy.

### Conclusions

This study validated the functional sensibility assessment. An ROC curve was constructed and the sensitivity, specificity, and optimal cutoff values were determined for the functional sensibility test with a precision pinch model. The functional sensibility results derived from the PHUA test could indicate CTS severity. A moderate correlation between the functional sensibility and traditional sensory tests suggests that this test could indicate the sensory function of the hand. Generally speaking, this novel test provides functional as well as practical perspectives on detecting deficit in both the sensibility and the precision pinch performance for patients with carpal tunnel syndrome. Further refinements in either the testing procedures or apparatus modifications are suggested improving the quality of functional sensibility test in the future works.

### Data Availability

The availability of data can be required.
